# Hybrid Surgery for Superior Mesenteric Vein Thrombosis: A Case Report

**DOI:** 10.31662/jmaj.2024-0305

**Published:** 2025-02-07

**Authors:** Ryota Sasaki, Takaaki Maruhashi, Marina Oi, Ayumi Takahashi, Kanako Okazaki, Yutaro Kurihara, Yasushi Asari

**Affiliations:** 1 Department of Emergency and Critical Care Medicine, Kitasato University School of Medicine, Sagamihara, Japan

**Keywords:** Acute, Abdomen, Thrombosis, Portal Vein, Mesenteric Veins

## Abstract

Superior mesenteric vein thrombosis (SMVT) is a rare condition characterized by thrombus formation in the superior mesenteric vein. SMVT is generally caused by abnormal blood coagulation, inflammation, or surgical interventions. This condition can lead to intestinal ischemia and necrosis due to blood flow stasis.

We report the case of a man in his 60s who presented with abdominal pain and vomiting. Abdominal contrast-enhanced computed tomography shows a thrombus in the portal and superior mesenteric veins, and reduced contrast in the small intestine. Approximately 1.5 m of the necrotic jejunum was resected, an open management approach was undertaken, and anticoagulation with continuous intravenous heparin was initiated. On the fourth day of treatment, a thrombus was retrieved from the superior mesenteric vein within the main trunk of the portal vein using a stent clot retrieval device. The patient’s bowel edema improved soon thereafter.

This case of SMVT was successfully managed using a hybrid approach of bowel resection and transcatheter thrombus retrieval.

## Introduction

Superior mesenteric vein thrombosis (SMVT) is a rare condition in which a thrombus forms in the superior mesenteric vein because of abnormal blood coagulation, inflammatory disease, or surgery ^(1)^. Idiopathic cases of SMVT are particularly uncommon ^(2)^. The condition can cause intestinal ischemia and necrosis resulting from blood flow stasis, with an acute mortality rate reported to range from 20% to 32% ^(1)^. Here, we present a unique case of idiopathic SMVT presenting with intestinal ischemic necrosis, which was successfully treated using a hybrid surgical approach combining necrotizing bowel resection and endovascular thrombus retrieval therapy under an open abdominal cavity.

## Case Report

**Figure 1.**
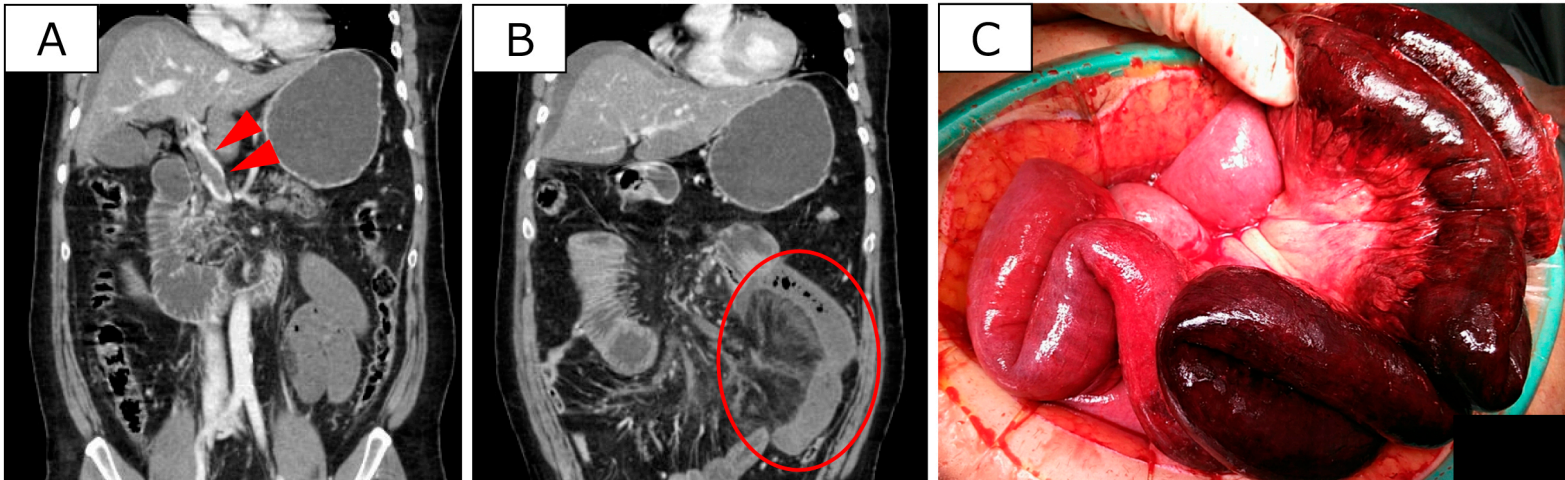
Contrast-enhanced computed tomography (CT) images upon arrival and intraoperative findings. (A) Coronal view showing an extensive thrombus in the main trunk of the portal vein (arrowhead). (B) A poor contrast area within a section of the small intestine accompanied by intestinal wall edema and thickening (circles). (C) Intraoperative findings showing bloody ascites, extensive necrosis of approximately 150 cm of the jejunum, and mesenteric edema.

**Figure 2.**
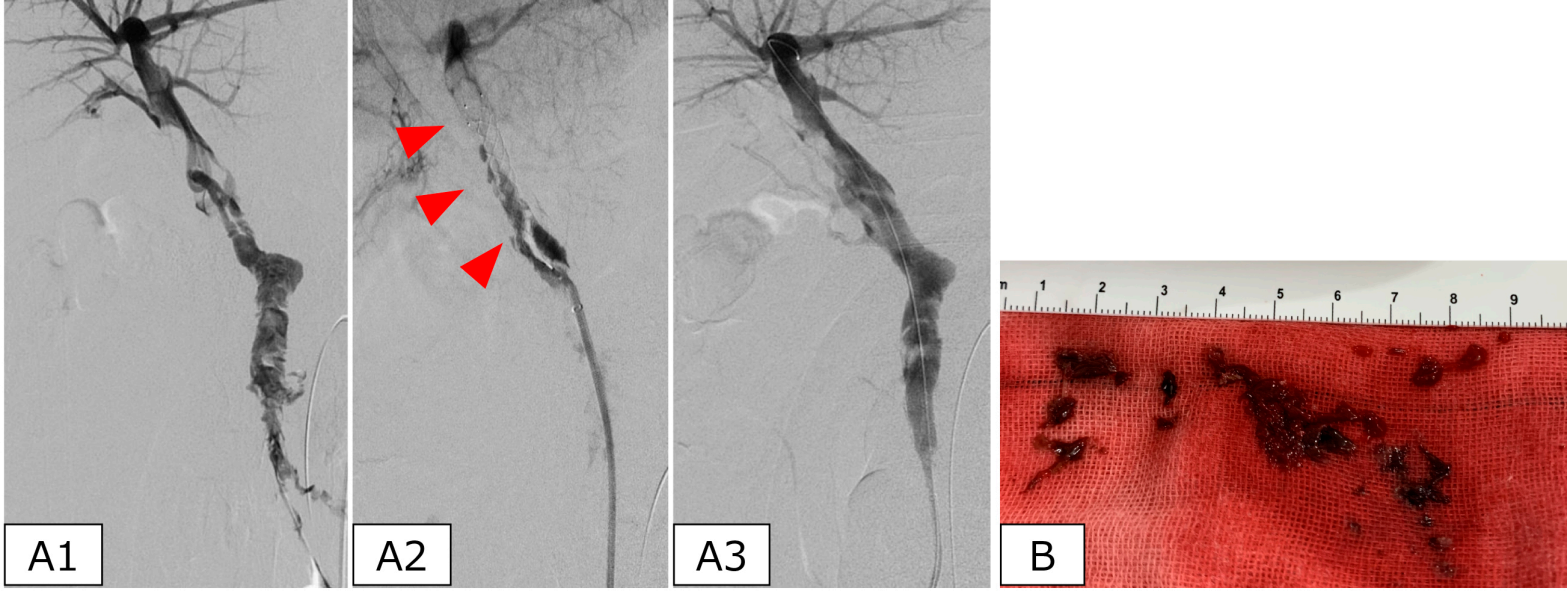
Endovascular thrombectomy of the portal vein. (A1) Superior mesenteric venography shows a diffuse contrast defect. (A2) Thrombus removal from the portal vein using a stent retriever (arrowhead). (A3) Confirmatory portography showing improved blood flow, with residual thrombus remaining. (B) The large thrombus volume is removed using the stent retriever.

The patient was a man in his 60s with a history of hypertension and dyslipidemia. He was rushed to our hospital with chief complaints of persistent abdominal pain and vomiting lasting for several days. Upon arrival, he was conscious, with vital signs showing a heart rate of 140 beats/min, blood pressure of 92/52 mmHg, and body temperature of 36.7°Ｃ. Blood tests revealed elevated fibrinogen/fibrin degradation products, increased D-dimer, and renal dysfunction (Table 1). Contrast-enhanced abdominal computed tomography (CT) revealed thrombi in the portal, superior mesenteric, and splenic veins; poor contrast in portions of the small intestine; intestinal tract dilatation with wall edema; and ascites (Figure 1A and 1B). Given the diagnosis of extensive small intestinal necrosis caused by SMVT, emergency laparotomy was performed. The procedure revealed extensive continuous necrosis of approximately 150 cm of the jejunum (Figure 1C). The necrotic intestine was resected; however, due to significant intestinal edema from venous stasis, abdominal closure was challenging. Consequently, open abdomen management was performed without intestinal anastomosis. Postoperatively, anticoagulation therapy with continuous intravenous heparin was initiated. The second surgery on day 3 confirmed the absence of further intestinal necrosis, although mesenteric edema from the residual portal vein thrombus continued to hinder abdominal closure. Therefore, endovascular thrombectomy was performed on day 4 of treatment. The superior mesenteric vein was punctured under an open abdomen. Attempts at catheter aspiration failed to remove the thrombus. Therefore, a stent-type thrombus retrieval device (Trevo^Ⓡ^ NXT ProVue Retriever 6 mm; Stryker, Kalamazoo, MI, USA) was used off-label for portal vein thrombosis. The proposed method retrieved a significant amount of thrombi. Subsequent contrast imaging revealed a residual thrombus in the portal vein, prompting the transcatheter intraportal administration of urokinase (Figure 2A and 2B).

**Table 1.** Blood Test Findings upon Patient Arrival at Our Hospital.

Complete blood count			Chemistry		
WBC	34.5 × 10^3^	/μL	Alb	2.3	g/dL
RBC	4.13 × 10^6^	/μL	BUN	66.6	mg/dL
Hb	12.9	g/dL	Cre	4.76	mg/dL
Plt	37.3 × 10^4^	/μL	AST	15	U/L
**Coagulation**			ALT	11	U/L
PT	18.5	sec	ALP	91	U/L
APTT	39.1	sec	CK	315	U/L
FDP	45.5	mg/mL	CK-MB	5	U/L
D-dimer	13.6	μg/mL	Na	130	mEq/L
AT-Ⅲ	73	%	K	3.5	mEq/L
**Arterial blood gas (4L/min oxygenation)**			Cl	93	mEq/L
pH	7.38		Ca	7.5	mg/dL
pO_2_	219.3	mm Hg	CRP	23.93	mg/dL
pCO_2_	40	mm Hg	protein C activity	62	%
HCO_3_^−^	23.1	mmol/L	protein S activity	65	%
Lac	15.9	mg/dL	HIT antibody	(-)

WBC: White blood cell count, RBC: Red blood cell count, Hb: Hemoglobin, Plt: platelets, PT: Prothrombin time, APTT: Activated partial thromboplastin time, FDP: Fibrinogen/fibrin degradation products, ATⅢ: Antithrombin Ⅲ, Lac: Lactate, Alb: Albumin, BUN: Blood urea nitrogen, Cr: Creatinine, AST: Aspartate aminotransferase, ALT: Alanine aminotransferase, ALP: Alkaline Phosphatase, CK: Creatine kinase, CK-MB: Creatine kinase MB, Na: Natrium, K: Kalium, Cl: Chlorine, Ca: Calcium, CRP: C-reactive protein, HIT: Heparin-induced thrombocytopenia

**Figure 3.**
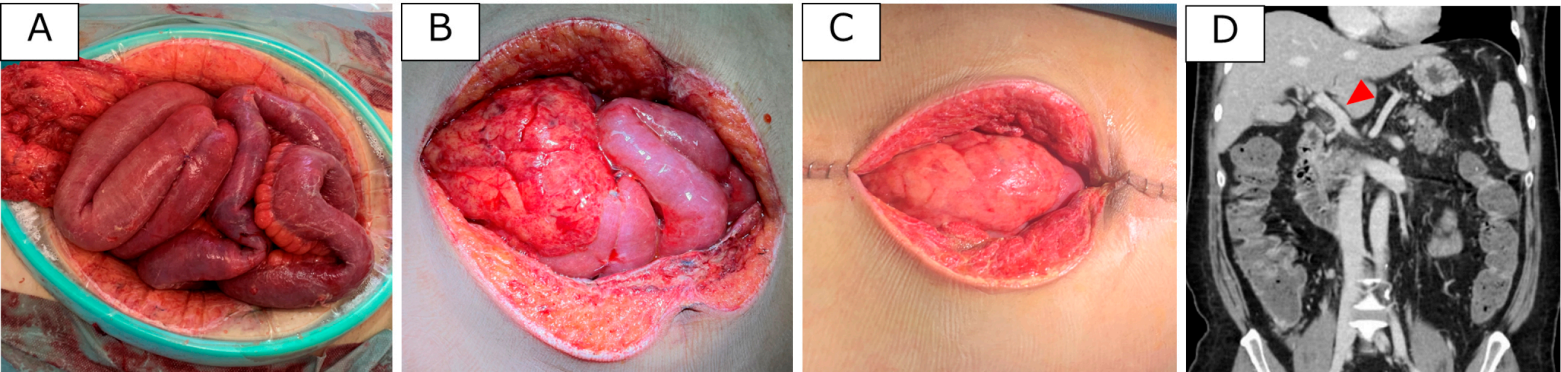
Bowel findings before and after thrombus retrieval. Condition of the open abdominal wound: (A) day 4, (B) day 6, and (C) day 11. Intestinal edema progressively improved, allowing for staged closure of the laparotomy wound. (D) Follow-up contrast-enhanced CT on day 42, showing resolution of the intraportal thrombus (arrowhead).

During the second-look surgery on day 3, small intestinal anastomosis was completed. Following endovascular treatment, the intestinal tract and mesenteric edema gradually subsided (Figure 3), allowing for abdominal closure on day 11. Follow-up CT on day 42 revealed near-complete resolution of the portal vein thrombus. The patient was then transferred to another hospital for rehabilitation on day 46.

## Discussion

In this case, the patient had no history of thrombogenic predispositions or underlying conditions that could induce a hypercoagulable state, recent surgery, trauma, or infection. In addition, coagulation tests revealed no abnormalities in factors such as protein C and S. Thus, SMVT was considered idiopathic.

The European Society for Vascular Surgery (ESVS) guidelines recommend immediate anticoagulation therapy and emergency surgery if intestinal necrosis is suspected upon the diagnosis of SMVT ^(3)^. If the thrombus-involved intestine remains, there is a risk of suture failure and recurrence ^(4)^. Moreover, extensive intestinal resection can cause short-bowel syndrome. To minimize necrosis, the ESVS guidelines also recommend direct incision of the portal vein to remove thrombi in patients with SMVT ^(3)^. However, endovascular thrombectomy was selected in this case due to the risk of bleeding associated with septic shock and the incision of the portal vein. The disadvantages of endovascular thrombectomy in the superior mesenteric vein include the high risk of bleeding with the percutaneous transhepatic portal vein approach ^(5)^. Direct puncture of the superior mesenteric vein may be a feasible approach for thrombus removal.

Although endovascular thrombectomy is often performed using a thrombus aspiration catheter, stent-type thrombectomy devices (stent retrievers) are also used in cerebrovascular interventions. These devices use a mesh tubular metal that entangles a blood clot when deployed from the catheter tip in the blood vessel, facilitating the removal of larger thrombi ^(6)^. Two factors likely contributed to incomplete thrombus removal in this case. First, only a 6.5-mm stent diameter was available. Second, veins contain fewer elastic fibers, making stent expansion difficult due to poor adhesion to the vessel wall. In other settings, large-diameter thrombus retrieval devices, such as the Flowtriever System (Inari Medical, Irvine, California), have been successfully used for pulmonary artery thromboembolism ^(7)^. In the future, these new devices with adequate stent diameters and materials that can be deployed and crimped to the vessel wall are expected to improve thrombectomy results in patients with SMVT.

Endovascular thrombectomy may be an effective approach for preventing intestinal necrosis in patients with SMVT.

## Article Information

### Conflicts of Interest

None

### Acknowledgement

The authors would like to thank Editage (https://www.editage.jp) for their assistance with the English language review.

### Author Contributions

Writing―original draft preparation, R.S. and M.O.; data curation, A.T. and K.O.; writing―review and editing, T.M. and Y.K.; supervision, Y.A. All authors have read and approved the final manuscript.

### Approval by Institutional Review Board (IRB)

The nature of this report was exempted from the requirement for institutional review board approval.

### Informed Consent

Informed consent for publication was obtained from the patient.
